# Assessment of Tissue Oxygenation and Radiation Dermatitis Pre-, During, and Post-Radiation Therapy in Breast Cancer Patients

**DOI:** 10.3389/fonc.2022.879032

**Published:** 2022-07-08

**Authors:** Edwin A. Robledo, Juan Murillo, Raquel Veiga Martin, Kevin Leiva, Corina Beiner, Maria Amelia Rodrigues, Marcio Fagundes, Joseph Panoff, Michael Chuong, Wensong Wu, Anuradha Godavarty

**Affiliations:** ^1^ Optical Imaging Laboratory, Department of Biomedical Engineering, Florida International University, Miami, FL, United States; ^2^ Department of Radiation Oncology, Miami Cancer Institute, Baptist Health South Florida, Miami, FL, United States; ^3^ Department of Mathematics and Statistics, Florida International University, Miami, FL, United States

**Keywords:** Tissue oxygenation, radiation therapy, radiation dermatitis, skin toxicity, near-infrared spectroscopy, breast cancer

## Abstract

Over 95% of breast cancer patients treated with radiation therapy (RT) undergo an adverse skin reaction known as radiation dermatitis (RD). Assessment of severity or grading of RD is clinically visual and hence subjective. Our objective is to determine sub-clinical tissue oxygenation (oxygen saturation) changes in response to RT in breast cancer patients using near-infrared spectroscopic imaging and correlate these changes to RD grading. A 4-8 week longitudinal pilot imaging study was carried out on 10 RT-treated breast cancer patients. Non-contact near-infrared spectroscopic (NIRS) imaging was performed on the irradiated ipsilateral and the contralateral breast/chest wall, axilla and lower neck regions before RT, across the weeks of RT, and during follow-up after RT ended. Significant changes (p < 0.05) in oxygen saturation (StO_2_) of irradiated and contralateral breast/chest wall and axilla regions were observed across weeks of RT. The overall drop in StO_2_ was negatively correlated to RD scaling (in 7 out of 9 cases) and was higher in the irradiated regions when compared to its contralateral region. Differences in the pre-RT StO_2_ between ipsilateral and contralateral chest wall is a potential predictor of the severity of RD. The subclinical recovery of StO_2_ to its original state was longer than the visual recovery in RD grading scale, as observed from the post-RT assessment of tissue oxygenation.

## Introduction

Breast cancer has become one of the most prominent diseases among women in the U.S. and one of women’s leading cause of death (1). The American Cancer Society has estimated that a total of 1.9 million new cancer cases will arise in 2022, 15% of which are breast cancer (1). Treatment for breast cancer includes lumpectomy or mastectomy in order to remove cancerous tissue from the breast. These procedures may then be followed by radiation therapy (RT) and/or chemotherapy in order to eradicate any microscopic residual malignant tissue and avoid recurrence of the disease. In most cases of RT, patients generally develop adverse skin reactions, one of the most prominent being radiation dermatitis (RD).

Radiation dermatitis is a side effect of RT whereby radiation that targets cancer cells inflicts collateral damage to nearby normal skin cells. RD manifestations can be as minor as reddening of the skin (erythema) or as severe as skin ulcerations and necrosis. Currently clinicians visually assess skin tissue health throughout patients’ RT based on the Common Terminology Criteria for Adverse Events (CTCAE) and/or Radiation Therapy Oncology Group (RTOG) grading scales. Currently there is no Level 1 evidence for any product used to treat radiation dermatitis.

Many research groups have performed different optical imaging studies to assess the physiological effect of RT on the irradiated tissues, *via in-vivo* human and animal studies for various cancer types as given in **Table 1**. The purpose of these studies is to objectively assess skin toxicity based on skin tissue oxygenation and/or hemoglobin concentrations. These objective assessments can complement the subjective visual assessment used by clinicians. Across the studies in head and neck cancers or breast cancer models, there is a correlation between hemoglobin concentrations and perfusion changes with the development of RD at the irradiated tissue site (2, 5–9, 13–15). While most of the studies focused on the impact of RT on the irradiated tissue, there has been no study that focused on the physiological effect of RT on the surrounding tissues nor their contralateral non-irradiated tissues. Hence, the objective of this study was to derive correlations between tissue oxygenation and skin toxicity in not only irradiated tissues, but also the surrounding tissues and its contralateral non-irradiated counterparts, in breast cancer patients undergoing RT. A non-contact near-infrared spectroscopy (NIRS) based imaging approach was used to perform weekly imaging during the 5-7 week RT.

**Table 1:** Optical imaging studies related to radiation dermatitis.

Optical Method	Contact-Based	Wavelength(s) nm	Human/Animal	Cancer Type	Measured Parameter(s)
DRS (2)	✓	500-700	Human	Head & Neck	Total Hemoglobin Concentration, Oxy- and Deoxy- hemoglobin
DRS (3, 4)	✓	450-650	Animal	✗ (only erythema)	Hemoglobin Concentration, Oxygen Saturation, Scattering Power
DCS (5)	✓	785	Human	Head & Neck	Relative Blood Flow (rBF)
LDI/LDF (6, 7)	✗	670	Human	Breast	Perfusion
HSI (8, 9)	✗	450-800	Human	Skin, Breast	Absorbance Spectra, Oxy- and deoxy-hemoglobin concentration, Perfusion
HSI (10, 11)	✗	500-660	Animal	✗ (regular irradiated skin)	Hemoglobin Oxygenation (sO2), Oxy- and deoxy-hemoglobin concentration, Perfusion
NIRS (6, 12)	✓, ✗	833-2,500	Human	Breast	NIR Reflective Spectra, Spatial-temporal changes in tissue oxygenation
SFDI (13)	✗	400-900	Human	Breast	Tissue Oxygenation Saturation, Total Hemoglobin Concentration, reduced scattering coefficients

DRS, diffuse reflectance spectroscopy; DCS, diffuse correlation spectroscopy; LDI, laser doppler imaging; LDF, laser doppler flowmetry; HSI, hyperspectral imaging; NIRS, near-infrared spectroscopy; SFDI, Spatial Frequency Domain Imaging.

## Materials and Methods

### NIRS Imaging Device

A non-contact NIRS imaging system, known as Snapshot_NIR_ (Kent Imaging, Inc, Calgary, CA), was employed to generate static 2D tissue oxygenation maps of the tissue. The system utilizes LEDs of multiple wavelengths (670, 730, 890, and 940 nm) within the red-NIR spectrum (650-1000 nm) to acquire diffuse reflectance images of the tissue and use them to compute tissue oxygenation measurements in terms of oxygen saturation (StO_2_). The device utilizes red lasers as positional indicators during imaging as well as fix the distance between the imaging plane and the device. The device contains a black box feature to correct the StO_2_ measurements obtained from patients with darker skin, i.e. high melanin concentrations. The NIRS device also acquires digital white light images within the field of view as the tissue oxygenation maps (i.e. 15 × 20 cm) and resolution (960 × 640 pixels). The measurements were acquired by the same team throughout the entire study to minimize operator error.

### Patient Recruitment/Information

This study was WIRB approved and was carried out at the Miami Cancer Institute (MCI). Ten (non-metastatic) breast cancer patients were recruited as they received a 4-8 week radiation therapy (RT) treatment. Prior to imaging, patients provided a written consent as well as HIPAA authorization to access their medical records related to the study.

Each patient was prescribed 42.6-50.4 Gy in 16-28 fractions. Only 3 of the 10 patients (patients 6, 7, and 9) received an extra radiation boost (10 Gy) towards the end of their RT. Two of the patients were black non-Hispanic (patients 4 and 10) while the rest were white Hispanic or non-Hispanic. Four of the 10 patients (patients 6-9) had a lumpectomy surgery prior to RT while the rest (patients 1-5) had a mastectomy. Eight patients received photon therapy and two received proton therapy. Patients 3-6, 9, and 10 were below 60 years and patients 1, 2, 7, and 8 were ≥60 years of age. Details of each recruited patient and their and treatment plan is given in **Table 2**.

**Table 2:** Detailed history of each recruited patient’s demographics, surgical procedure, tumor location, radiation treatment plan, and dosage.

Case	Age	Demographics	Surgery	Location of Tumor	Right/Left	Proton/Photon	Total Dose (Gy)
1	61	White Hispanic	Mastectomy	12 o’clock	Left	Photon	50.4
2	76	White Hispanic	Mastectomy	12 o’clock	Left	Proton	50.4
3	56	White Non-Hispanic	Mastectomy	2 o’clock	Right	Photon	50.4
4	57	Black Non-Hispanic	Mastectomy	12 o’clock	Right	Photon	50.4
5	49	White Non-Hispanic	Mastectomy	12 o’clock	Left	Photon	50
6	47	White Hispanic	Lumpectomy	3 o’clock	Left	Proton	50.4 + 10 Boost
7	77	White Non-Hispanic	Lumpectomy	7 o’clock	Right	Photon	42.56 + 10 Boost
8	68	White Non-Hispanic	Lumpectomy	12 o’clock	Right	Photon	42.56
9	40	White Non-Hispanic	Lumpectomy	9 o’clock	Right	Photon	42.56 + 10 Boost
10	34	Black Non-Hispanic	Mastectomy	12 o’clock & 4 o’clock	Left	Photon	50.4

### Imaging Approach

Imaging of patients was performed weekly over the full duration of their RT, with a follow-up imaging session 4 weeks after the completion of RT. Week-0 of imaging was performed prior to the beginning of the RT (i.e. pre-RT) on all recruited patients. Each weekly imaging session was performed immediately following the clinician’s assessment of RD but before the patient’s scheduled RT procedure. Patients were imaged at an angled sitting position. Images were attained from several regions of breast tissue and its surroundings that were possibly affected by RT (as typically observed by the clinicians).

These regions included the chest wall, lower neck, and axilla (underarms). Each region was imaged 3-4 times per session, at slightly varying angles. Each repeated image represented a repeated trial for quantitative and statistical analysis. Both the ipsilateral irradiated areas and their contralateral non-irradiated tissue were imaged at all the regions (chest wall, lower neck and axilla). Several fiducial markers were placed to obtain an approximate spatial reference across weeks of imaging the same patient. The key fiducial markers were at the inner ends of the clavicle, and close to the nipple region of both the breast tissues. Pre-RT imaging studies were performed to determine correlation of inherent tissue oxygenation differences between the irradiated and the non-irradiated breast tissues on the severity of RD as assessed visually. A one-month post-RT images were also acquired to determine if the rate of tissue recovery from RD was visually similar to the physiological change in terms of tissue oxygenation.

### Pre-Analysis: Segmentation and ROI Selection

The diffuse reflectance signals obtained during imaging studies were automatically processed by the built-in software of the device to obtain tissue oxygenation (StO_2_) maps. A manual freehand segmentation technique combined with edge detection was performed on these StO_2_ maps to isolate only the region of the chest wall within the plane of focus of the device and remove the irrelevant surrounding tissues. The fiducial markers were also segmented out using this technique. Segmentation was not applied to StO_2_ maps of the lower neck and axilla.

**Figure 1:**
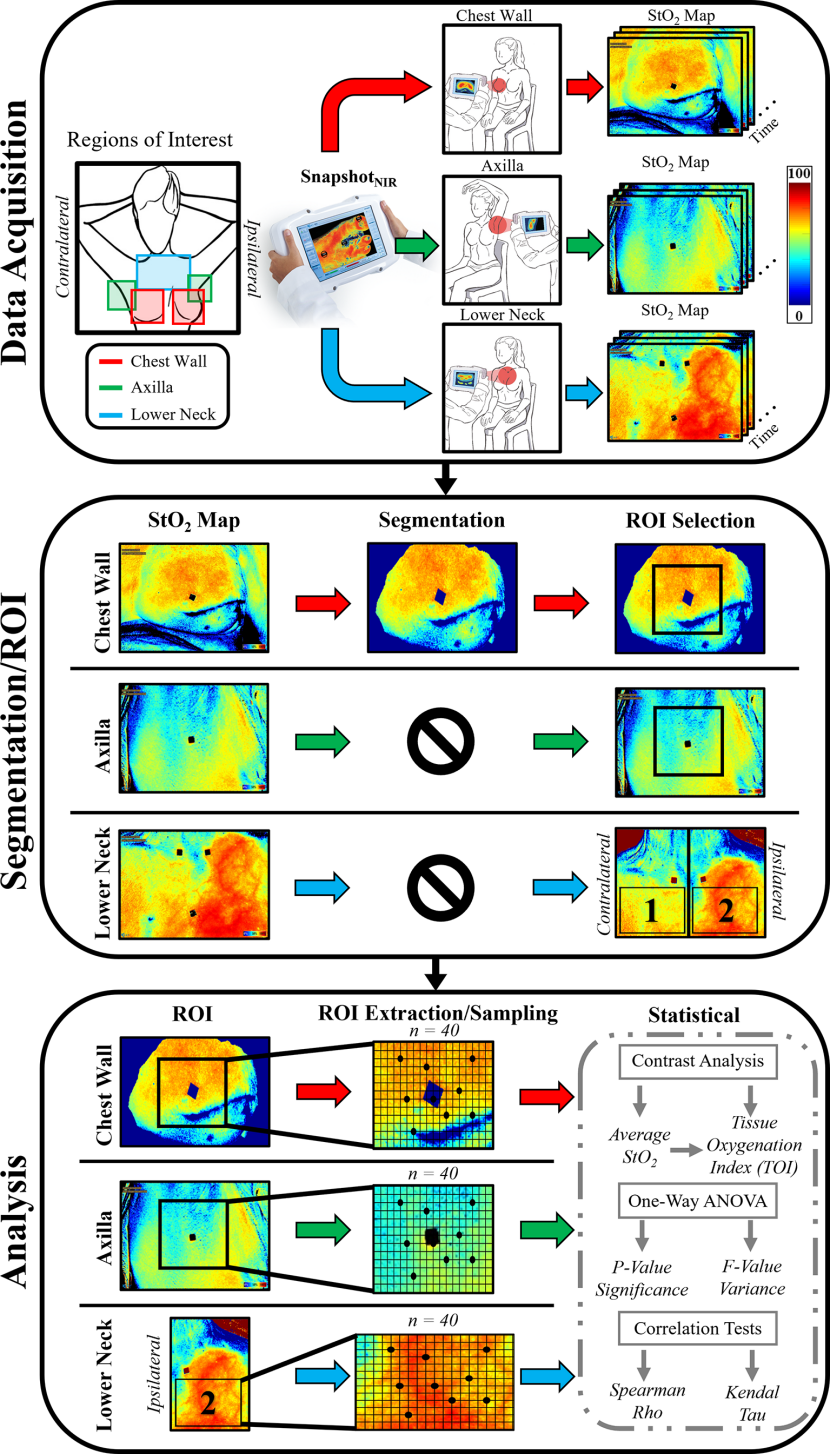
Schematic demonstrating the workflow and methodology of the study.

A square region of interest (ROI) was extracted from each segmented (chest wall) or the non-segmented (lower neck and axilla) StO_2_ images (as shown in **Figure 1**). A 300 × 300-pixel resolution ROI was extracted from the chest wall and axilla, centered on a fiducial marker. A large ROI oriented by the fiducial markers on the clavicle ends and sternum was extracted from the lower neck.

Within the extracted ROIs, 40 sample points were randomly selected for statistical relevance. The sample points were used toward further analysis.

### Study A: Effect of RT on Tissue Oxygenation Across Weeks of RT

Analysis of longitudinal changes in ipsilateral and contralateral tissues across chest wall, axilla, and lower neck during RT was done using the sampled ROI data points. The StO_2_ measurements across the ROI were averaged for 3 trials and plotted (with error bars) across time of the RT. A contrast of the average StO_2_ from each week with respect to average StO_2_ from the baseline (week 0) was determined as the tissue oxygenation index (TOI), as given in Equation 1. The TOI values were plotted across time of treatment.


(1) $$TOI\left(t\right)=\frac{{\left({\text{StO}}_{2}\right)}_{\text{Avg},\text{Week}(\text{t})}-{\left({\text{StO}}_{2}\right)}_{\text{Avg},\text{Week}(0)}}{{\left({\text{StO}}_{2}\right)}_{\text{Avg},\text{Week}(0)}}\times 100\%$$

A one-way ANOVA (α = 0.05, 95% confidence) was carried out to determine if the extent of changes in StO_2_ during RT were significant. The test was applied to each trial individually. The p-value was used to determine if the changes were significant, and the F-value was used to determine the relative extent of StO_2_ changes across RT when comparing across different cases.

### Study B: Correlation of RD to Tissue Oxygenation

The development of RD was compared to tissue oxygenation 1) pre-RT, 2) during RT, and 3) post-RT.

#### Study B.1: Evaluating pre-RT StO_2_ Contrast and Comparing to Max RD Grading

The focus of this study was to determine if the baseline (pre-RT) StO_2_ differences between the ipsilateral and contralateral chest wall has any correlation with the maximum grade of RD development by the patient during their course of the RT. This physiological information can potentially determine if patients are at risk for severe RD. The pre-RT StO_2_ contrast between the ipsilateral and contralateral chest wall regions were calculated based on Equation 2. The contrast was then compared to the maximum RD grading scale.


(2) $$Contrast_{pre-RT}=abs\left(\frac{{\left({\text{StO}}_{2}\right)}_{Irr,\;Week\left(0\right)}-{\left({\text{StO}}_{2}\right)}_{Nonirr,\text{ Week}\left(0\right)}}{{\left({\text{StO}}_{2}\right)}_{Nonirr,\text{Week}(0)}}\right)\times 100\%$$

#### Study B.2: Evaluating Changes in StO_2_ During RT and Comparing to Changes in RD

The focus of this study was to derive correlations (if any) between the development of RD and changes in StO_2_ across weeks of RT. This could provide evidence toward association between the two parameters (StO_2_ and RD). The changes in average StO_2_ of the ipsilateral (irradiated) tissues were compared to the development of RD during RT. Due to the ranked data from RD CTCAE grading scale and the discrete data from the average StO_2_ measurements, nonparametric correlation methods were utilized. One of the statistical methods used was Spearman’s Rho (14, 15), which was used to determine direction and strength of association between the two parameters. Rho was calculated using Equation 3 and ranges from -1 (negative correlation) to 1 (positive correlation).


(3) $$\rho \left(StO_{2},RD\right)=1-\frac{6\sum d^{2}}{n\left(n^{2}-1\right)}$$

The second statistical method used was Kendall’s tau (14, 15), which indicates if two parameters are statistically dependent. Tau was calculated using Equation 4 and have the same range and outcome as Spearman’s Rho.


(4) $$\tau \left(StO_{2},RD\right)=\frac{2K}{n\left(n-1\right)}$$

The combination of both methods can provide evidence toward the correlation of StO_2_ and RD during RT.

#### Study B.3: Evaluating Post-RT StO_2_ Contrast Between Last Week vs Follow Up

The focus of this study was to determine if the StO_2_ changes between post-RT assessment (typically during 1-month follow-up) and last week of RT correlated to the visual reduction in RD grading during the follow-up visit. This could provide clinicians additional sub-clinical information to better assess the healing of tissues affected by irradiation after RT ends. The contrast between average StO_2_ of the last week of RT and follow-up assessment of the irradiated chest wall was calculated, as given by Equation 5.


(5) $$Contrast_{post-RT}=\frac{{\left({\text{StO}}_{2}\right)}_{Irr,\;1-month}-{\left({\text{StO}}_{2}\right)}_{Irr,\text{ Week}\left(\text{last}\right)}}{{\left({\text{StO}}_{2}\right)}_{Irr,\text{Week}\left(\text{last}\right)}}\times 100\%$$

The post-RT contrast was calculated for the ipsilateral chest wall and compared to the change in RD CTCAE grading between the last week of RT and follow-up assessment. The axilla and lower neck were not graded for RD at the follow-up assessment and omitted from post-RT analysis.

## Results

### Study A: Effect of RT on Tissue Oxygenation Across Weeks

**Figure 2:**
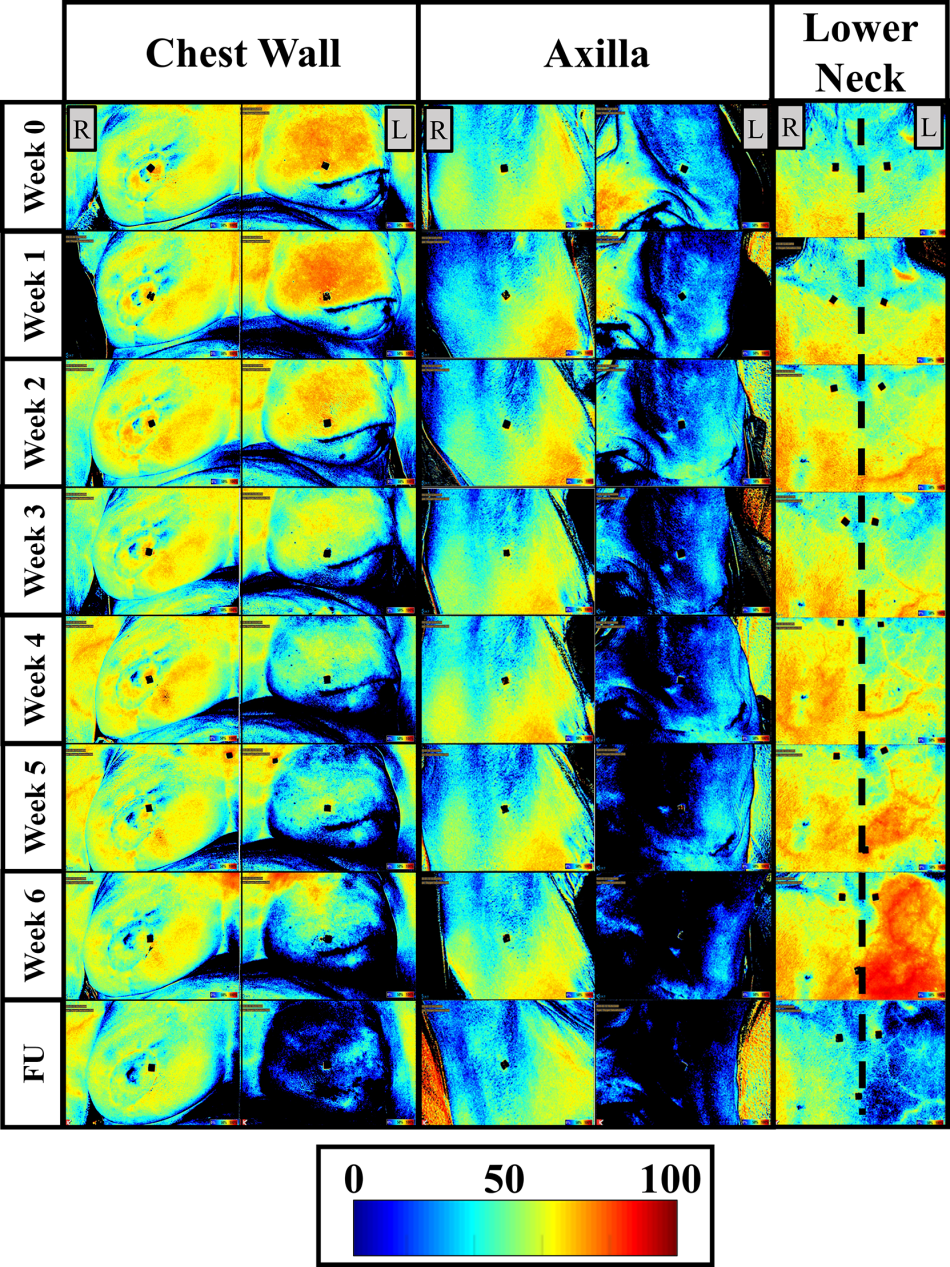
Two-dimensional pseudo color maps of StO_2_ of the ipsilateral (L) and contralateral (R) chest wall, axilla, and lower neck across weeks of RT and a follow-up (FU) after RT finished (1-month FU) for patient 2. Week 0 was acquired before any RT was carried out and is considered the baseline/reference image. Colorbar represents the percentage of StO_2_ ranging from 0-100%.

The oxygen saturation (StO_2_) maps were obtained from all patients and across all weeks of imaging, and a sample case is shown in **Figure 2**. A 2D pseudo-color map of StO_2_ of the ipsilateral and contralateral chest wall, axilla, and lower next region of patient 2 (from **Table 2**) across week 0-6 and 1-month follow-up is shown in **Figure 2**. In the 2D StO_2_ maps, red indicates high concentrations of oxygen saturation and blue indicating low concentration of oxygen saturation.

#### Qualitative Analysis of StO_2_ Changes

A distinct difference in the StO_2_ maps was observed in the ipsilateral regions of the chest wall, axilla, and lower neck across the weeks of RT and in comparison to their respective contralateral tissues. While the StO_2_ with RT in the chest wall and axilla regions, a decreasing trend was not distinct in the lower neck region.

No distinct differences in the StO_2_ maps were observed in the contralateral chest wall, axilla, and lower neck regions of patient 2 across weeks of RT. These StO_2_ maps from the contralateral tissues detected relatively minimal changes across weeks of RT, compared to that in the ipsilateral tissues. This trend was also seen in most patients. A majority of patients (patients 1, 3-5, 7-9) demonstrated a distinct decrease in the StO_2_ in both the contralateral and ipsilateral tissues, although it was to a greater extent in the ipsilateral tissue. This indicates that the contralateral regions were exhibiting changes in StO_2_ in response to RT, although not to a high extent as the ipsilateral tissue.

#### Quantitative Analysis of StO_2_ Changes

**Figure 3:**
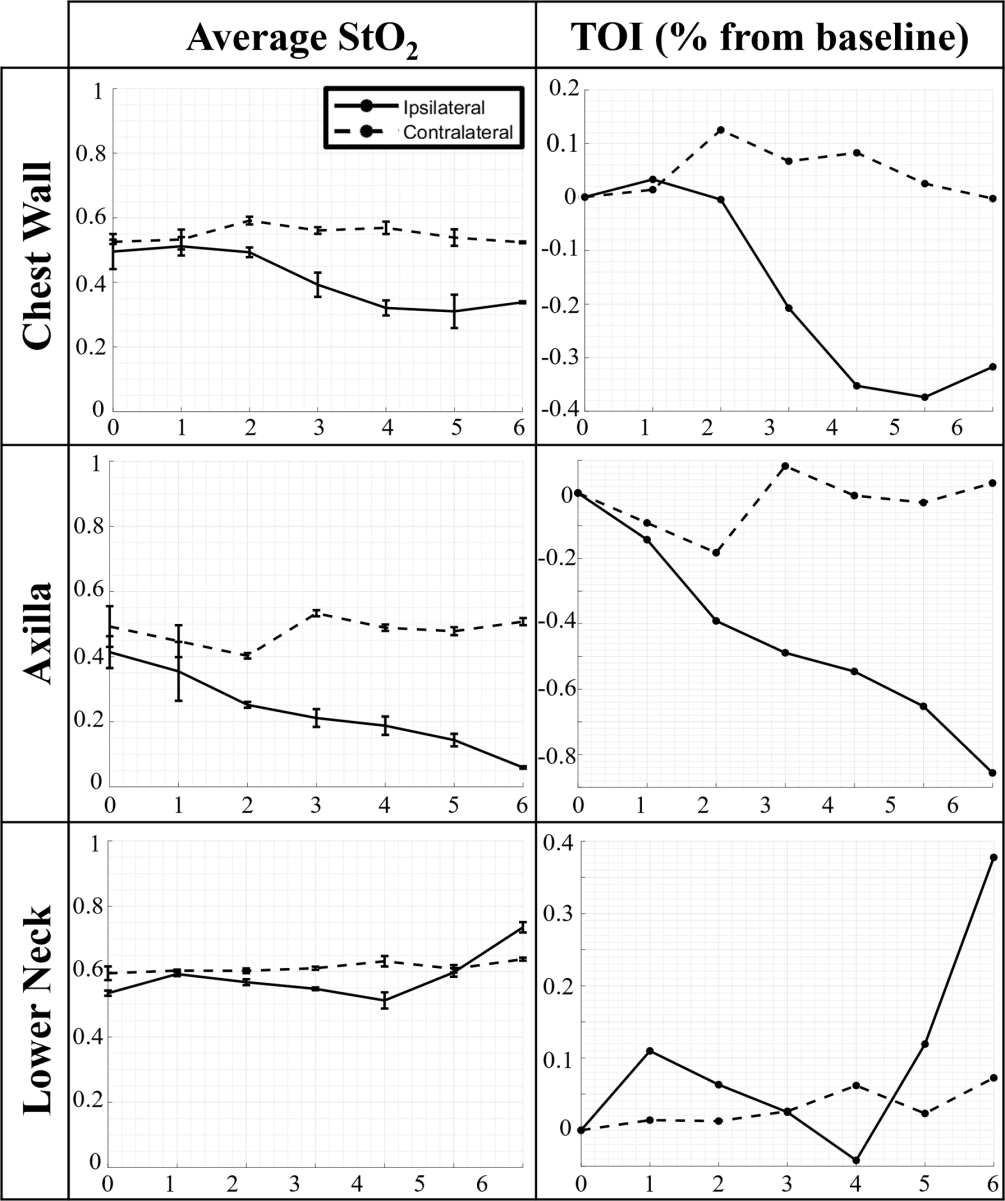
Changes in average oxygen saturation (StO_2_) and tissue oxygenation index (TOI) across weeks of RT for every imaged region (chest wall, axilla, and lower neck).

The average StO_2_ and TOI across weeks of RT from patient 2 is shown in **Figure 3** A one-way ANOVA analysis was performed across all patients, their weeks of RT, and all 3 trials to determine if the changes in average StO_2_ across RT were significant across the imaging time points. The average of the p-values from the three one-way ANOVAs across each trial were further compiled across all patients and imaged regions (ipsilateral and contralateral chest wall, axilla, and lower neck) to determine if the changes in average StO_2_ across weeks of RT were significant (i.e. p<0.05).

From analysis of the ipsilateral data, all patients’ irradiated chest wall region and a majority of patient’s irradiated axilla region (n=8) expressed a significant change (p<0.05) in average StO_2_ throughout RT. This is consistent with past work, demonstrating that there are changes in hemoglobin parameters throughout RT, especially demonstrating changes in oxyhemoglobin (HbO) during radiation exposure (9). The changes evaluated in the ipsilateral chest wall and axilla were noted to mostly have a negative trend in average StO_2_ across time of RT, similar to the case in **Figure 3**. This negative relationship is more distinct when plotted as a contrast parameter (TOI). It has been documented in literature that during continued radiation therapy, patients experience symptoms including improper vascularization and decreased blood perfusion due to damage in the endothelium (16). Within the ipsilateral chest wall and axilla regions, it was observed that several patients (n=4) expressed an initial increase in average StO_2_ before decreasing thereafter. This can be representative of the initial response of inflammation due to RT (16, 17). It was also noted from previous studies that tissue oxygenation increased as an early response to tissue irradiation (4, 10). All patient’s lower neck regions expressed no significant change in average StO_2_ throughout RT.

From the statistical analysis of the contralateral tissue, a majority of the patients’ chest wall region (n=9) and axilla region (n=8) expressed a significant change (p<0.05) in average StO_2_ across weeks of RT. This further indicates that radiation from RT is having an impact on the contralateral chest wall and axilla regions, apart from the irradiated ipsilateral regions. The impact can be caused by radiation spill from the proton or photon RT received by the patients (18, 19). Radiation spill can launch stray ionizing particles to come in contact with tissues not targeted for therapy. Although statistically a significant change was found, the contralateral chest wall, axilla, and lower neck demonstrated no distinct consistent trend (an increase or decrease in oxygenation across the weeks of treatment) among most of the patients, similar to what is shown in **Figure 3**. This is evidenced further by the TOI plots for the contralateral chest wall, axilla, and lower neck which indicates that most patients’ extent of change in average StO_2_ were smaller than their counterpart ipsilateral regions.

**Figure 4:**
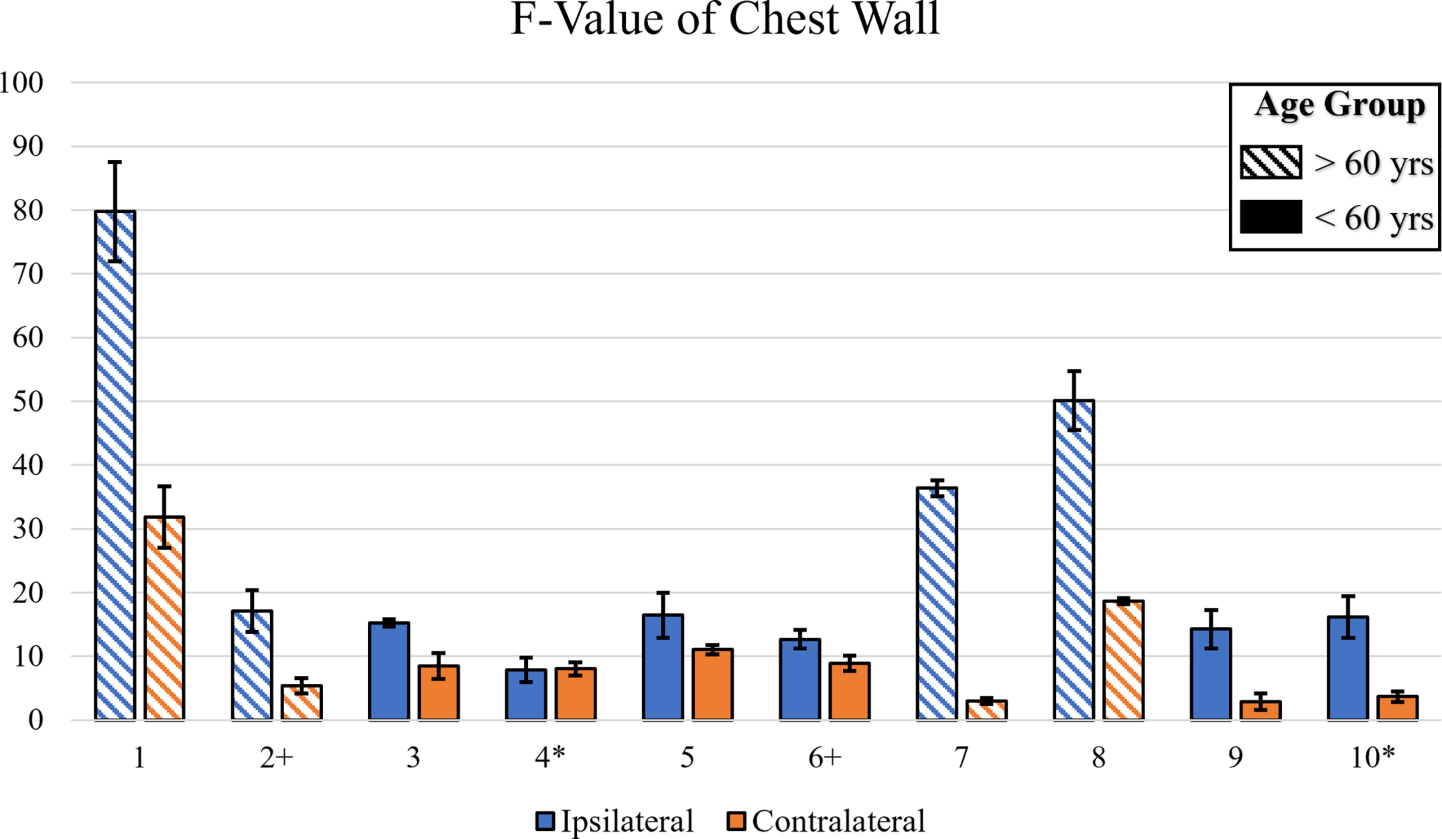
A bar plot illustrating the F-value across all patients for the irradiated chest wall. The F-value was calculated *via* One-Way ANOVA for each trial. Symbols near patient number indicate dark skin (*) or ≥60 years old (+).

The F-value from the one-way ANOVA across all patients’ chest wall were plotted in **Figure 4**. The data was categorized by age group and ipsilateral or contralateral region. The F-values for ipsilateral regions were generally higher compared to contralateral regions, indicating a greater extent of change in average StO2 in irradiated ipsilateral regions compared to the contralateral regions. It was also noted that the F-values chest wall and axilla produced higher F-values as compared to the lower neck region for most patients (not shown for brevity). This indicates that the extent of change in average StO_2_ in the ipsilateral chest wall and axilla is greater than the lower neck. Additionally, a relatively high F-value were evaluated for patients 1, 2, 7, and 8 in the chest wall region, whose ages ranged between 61-77 years. This indicates the extent of change of average StO_2_ in response to RT is greater in older patients (≥ 60 years). This can be from thinning of the skin with age, especially in post-menopause women (20). The thinner skin provides less protection toward radiation.

### Study B: Correlation of RD to Tissue Oxygenation

All the RD CTCAE gradings observed and recorded by the clinicians during RT on the same day of each imaging session are displayed in **Table 3**.

**Table 3:** The CTCAE RD gradings at each imaging session for each patient is obtained from the clinician.

Patient	Pre-RT Surgery M/L	RD before and during RT	RD post-RT (*3-Mo.)
1+	M	(0,0,1,1,1,2)	1
2+	M	(0,0,0,1,1,2,2)	0
3	M	(1,1,2,2)	0
4*	M	(0,0,0,0,0,0)	0*
5	M	(0,0,0,1,2,2)	0
6+	L	(0,0,1,0,1,1,1,1)	0*
7+	L	(0,1,1,2,2)	1
8+	L	(0,0,1,1)	1
9	L	(0,0,1,2)	0
10*	M	(0,0,0,0)	0*

Each patient underwent surgery before RT, which was either mastectomy (M) or lumpectomy (L). Symbols near patient number indicate dark skin (*) or ≥60 years old (+).

Most patients developed radiation dermatitis (RD) during RT. The most severe cases of RD grading reached grade 2, which occurred in 6 out of 10 patients. In patients 4 and 10, there were no changes in RD grading throughout the RT. However, imaging of patient 10 was interrupted after 4 sessions of StO_2_ measurements due to the pandemic (COVID-19), although her RT continued and she developed RD. Patient 6 was the only patient that was observed to have a decrease in RD grading during their RT. This may be because her RT was spaced out due to many missed treatment days, allowing her to recover intermittently. Patient 3 was the only patient to begin with an RD grading of 1, as she paused her initial RT and imaging from her first restart week was considered as her week-0 (with RD grading 1). It was noted by several patients that their condition worsened following the conclusion of their RT but was not observed nor recorded by a radiation oncologist for the grading scale until the standard one-month follow up visit.

#### Study B.1: Evaluating pre-RT StO_2_ Contrast and Comparing to Max RD Grading

**Figure 5:**
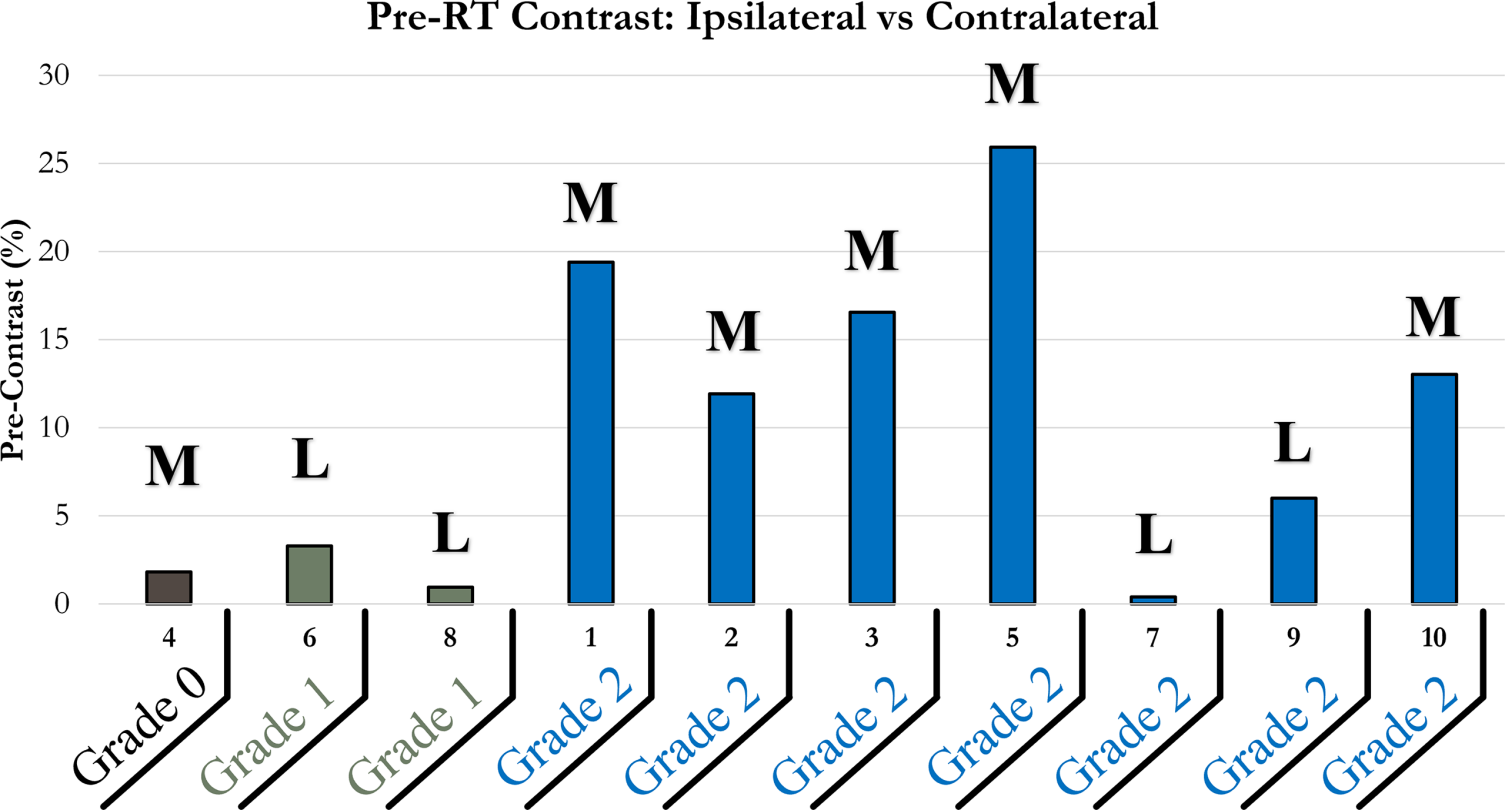
Bar plot of pre-RT StO_2_ contrast of ipsilateral and contralateral chest wall. Patients were grouped horizontally based on the maximum RD grading experienced during their RT; 0 (brown), 1 (green), 2 (blue). Bolded letters included above bars indicate mastectomy (M) or lumpectomy (L) surgical procedures performed prior to RT.

The contrast between the ipsilateral and contralateral StO_2_ were evaluated to determine if it could be a predictor of the severity of RD that the patient may experience due to the RT. The pre-RT StO_2_ contrast across the patients was calculated and shown in **Figure 5**. Data was categorized by the maximum RD grade experienced by patients during RT, and the nature of surgical procedure performed prior to RT.

It was apparent that a majority of patients (n=6) had a relatively higher pre-RT StO_2_ contrast (>5%). These relatively higher pre-RT StO_2_ contrast (>5%) correlated to all patients who developed RD grade 2. The difference between the contralateral tissue and the targeted ipsilateral tissue may indicate that physiologically the targeted chest wall had underlying abnormal tissue oxygenation (StO_2_). This is either attributed to poor vasculature or atypical perfusion. This can indicate that pre-RT tissue oxygenation in the targeted irradiated region may be an indicator of the potential severity in RD because of RT.

Consistent with the observation made regarding a high pre-RT StO_2_ contrast, it was also noted that a low pre-RT StO_2_ contrast could be an indicator for less severe RD during RT. Four (patients 4,6,7, and 8) out of the ten patients experienced a pre-RT StO_2_ contrast below 5%. Three out of these four patients (patients 6,7, and 8) experienced a maximum of RD grade 1 throughout the course of their RT. This further supports the correlation between pre-RT StO_2_ contrast and maximum RD grade during RT.

Additionally, from **Figure 5**, it was apparent that among the 6 patients who had received a mastectomy, 5 had a high pre-RT StO_2_ contrast (>5%) and experienced a max RD grade of 2. Among the 4 patients who had received a lumpectomy, 3 had a relatively low pre-RT StO_2_ contrast (<5%) and 2 had experienced a max RD grade of 1. This is congruent to what has been observed in the past, that patients that undergo mastectomy tend to experience more severe RD from RT, compared to lumpectomy patients (21, 22). Therefore, pre-RT tissue oxygenation based contrast can potentially be part of the contributing factors toward risk assessment to determine if a patient is pre-disposed to adverse reactions during RT.

#### Study B.2: Evaluating Changes in StO_2_ During RT and Comparing to Changes in RD

**Figure 6:**
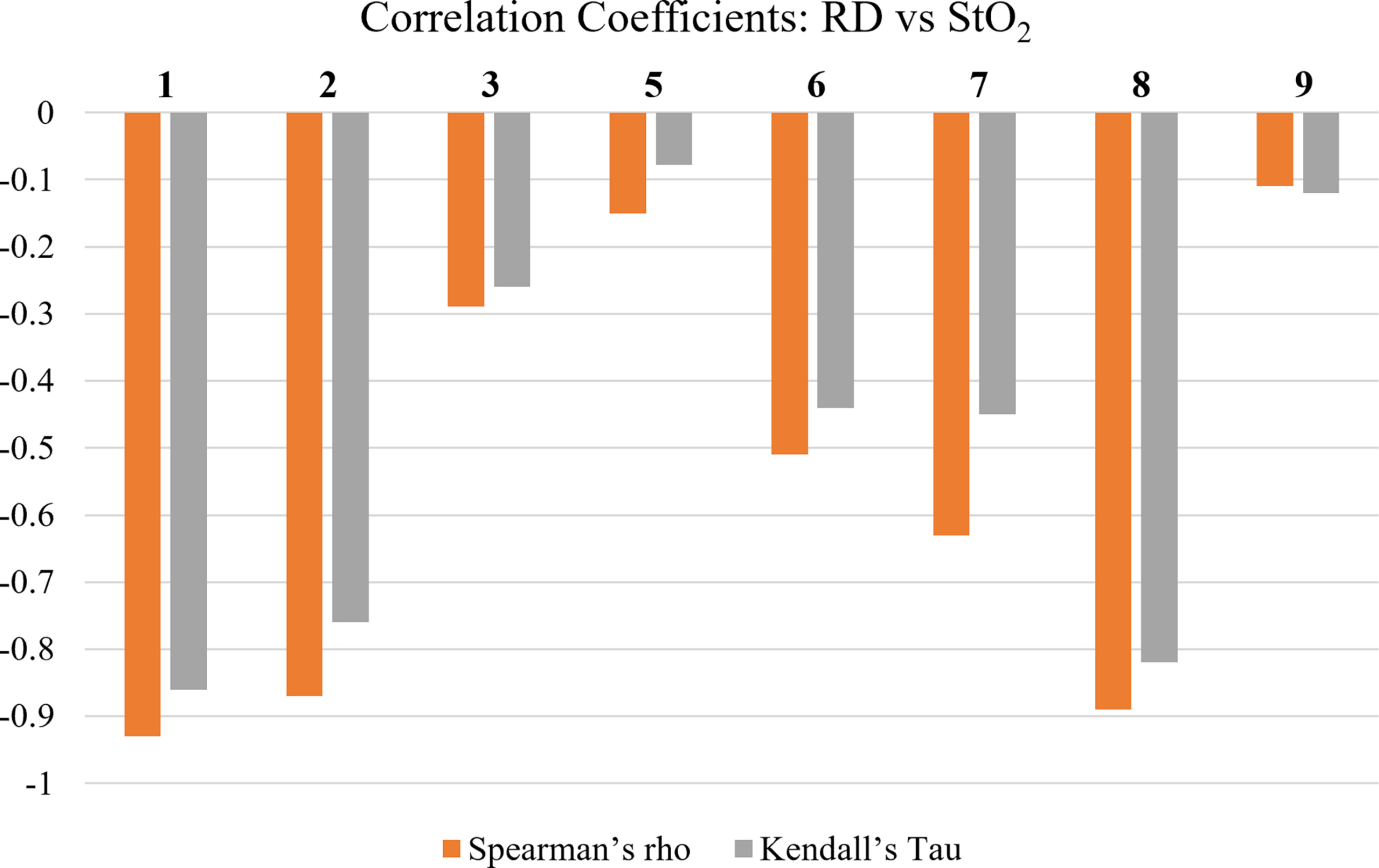
Spearman’s Rho’s and Kendall’s Tau’s correlation coefficients between average oxygen saturation (StO_2_) and RD grading. Patients 4 and 10 had no reported RD grading during RT and were therefore omitted from this analysis.

Statistical correlation tests were utilized to evaluate if the changes in RD grading had a relationship with the changes in tissue oxygenation (StO_2_) during RT. The correlation coefficients for Spearman’s Rho and Kendall’s Tau were evaluated and plotted in **Figure 6**.

The correlation tests demonstrate that in most patients there was a negative relationship between RD grading and changes in StO_2_. The negative correlation combined with the trend of StO_2_ found in study A (and **Figure 3** for sample patient 2) indicate that in most cases StO_2_ decreases as RD grading increases. This is consistent with past studies that have concluded similar findings (9). Although patient 4 produced no correlation due to no RD grade changes, it was evaluated in study 1 that they exhibited the least amount of StO_2_ change across time. This can be seen based on their low F-value from a one-way ANOVA, as shown in **Figure 4**. This further supports the relationship between the two parameters.

#### Study B.3: Evaluating Post-RT StO_2_ Contrast Between Last Week vs Follow Up

**Figure 7:**
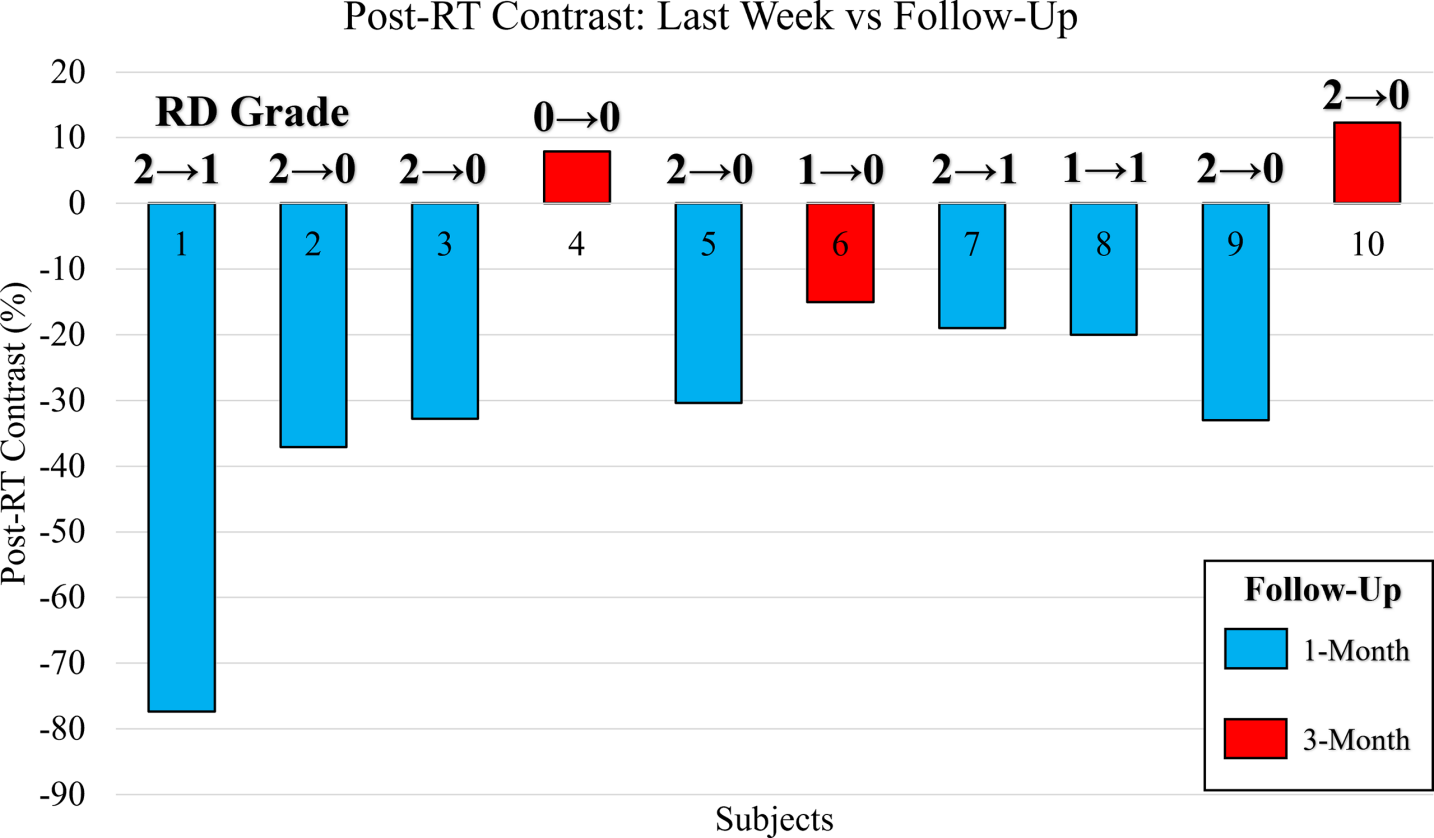
Bar plot of the post-RT StO_2_ contrast between the last week of RT and follow-up assessment. Contrast reported as a percentage change of the follow-up assessment with respect to the last week of RT. Bars color coded to indicate patients assessed at 1-month (blue) and 3-months (red). The CTCAE RD grading as provided by clinician is included for the last week of RT and during the follow-up assessment (RD_Last Week_ → RD_FU_).

A post-RT contrast was carried out between the StO_2_ measurements during the follow-up assessment after completion of RT and the last week of RT, and shown in **Figure 7**. Most patients had their follow-up assessment 1-month after their RT ended, but due to the pandemic (COVID-19), patients 4, 6, and 10 had a larger time period (3+ months) between the last week of RT and their follow-up assessment.

It was noted that in a majority of the patients, the RD grading decreased to a 0 or 1 by the follow-up assessment, while in 2 cases it remained the same (patients 4 and 8). All the cases where the follow-up assessment was 1-month from the last week of RT (n=7) displayed a relatively high negative post-RT StO_2_ contrast (<-18%). This may indicate that at the 1-month assessment, patients may still have underlying physiological abnormalities with respect to the StO_2_. In the cases (n=3) where the follow-up assessment occurred 3+ months after the last week of RT, a relatively higher post-RT StO_2_ contrast (mostly positive, and definitely >-18%). This could indicate that the patients’ underlying tissue oxygenation, which decreased with RT was increasing with time after completion of RT, indicating vasculature and tissue recovery of those affected regions at a slower pace than visual changes. Clinicians and patients have noted that in the weeks after RT concludes and before the follow-up assessment, patients continue to experience worsening adverse effects from RT. The adverse effects can be as severe as moist desquamation coupled with bleeding which indicates grade 3 RD. This may give evidence toward prolonged effects of RT and would indicate why high negative post-RT StO_2_ contrast was evaluated for patients at the 1-month follow-up assessment.

## Discussion

The effects of radiation therapy on skin toxicity has been extensively studied across the years (23). In more recent years, the physiological changes in tissue oxygenation in response to radiation therapy and its correlation to skin toxicity has been studied in *in-vivo* head and neck cancers and breast cancer patients and/or *via* animal studies (2–13). Tissue oxygenation was shown to change with the extent of skin toxicity, quantified by CTCAE grading scales for radiation dermatitis. While most of the past studies (as given in **Table 1**) have demonstrated that tissue oxygenation changed with extent of skin toxicity, the correlation of these changes across weeks of RT was not performed.

From our current studies, we observed that the tissue oxygenation (measured in terms of oxygen saturation) in many cases initially increased with the onset of RT, possibly from an inflammatory response of the skin to radiation. Upon continued RT, the overall oxygen saturation decreased across weeks and this drop correlated negatively with the severity of RD. The changes in physiology from RD have an impact on tissue oxygenation and how hemoglobin travels to certain regions of tissue. Inflammation is generally an early and common adverse reaction that is induced by RT. Inflammation is the body’s response to remove any foreign pathogens and isolate any injuries to a localized region by increasing vascular permeability with vasodilation. This allows neutrophils, monocytes, and nutrients to localize to the region (17). This results in an increased concentration of oxygen-bonded hemoglobin to the region to compensate for increased metabolic demand. Continued RT leads to skin deformation and damage to the endothelium. The irradiated area experiences improper vascularization, leading to less blood perfusion. This can cause a sharp decrease in hemoglobin concentration and other nutrients which results in a reduction in metabolic and healing processes. In more severe cases of RD, if inflammation persists, the skin is susceptible to atrophy or necrosis, causing further decrease in perfusion and oxygenated hemoglobin to the region. In most cases, the severity in RD is lesser (typically 2 or 3) in RT-treated breast cancer patients than in head and neck cancers because of lower delivered dose. From our studies, we observed that it is not only the irradiated chest wall region that is impacted by RT, but also the surrounding tissues such as the axilla and the lower neck regions that are exposed to radiation. There is a drop in tissue oxygenation in these surrounding regions apart from the breast/chest wall due to the extent of dose delivered to the region of interest. This confirms that there are changes in the underlying physiology from RT in the tissue surrounding the irradiated regions and from radiation spill, although the changes are not manifested as visual changes to record a RD grading scale. Additionally, the radiation spill and the mild radiation dosage given to the contralateral tissues also causes a (statistically significant) decrease in tissue oxygenation across weeks of treatment, although this drop is much less than that in the irradiated ipsilateral tissues. The contralateral tissues were not evaluated for RD grading by clinicians. Hence the clinical significance of this decrease in tissue oxygenation in the contralateral tissues is not known and can explored *via* a future study where clinical evaluation of contralateral tissues are also obtained.

Approximately 95% of the patients will develop skin erythema and will manifest RD of different grading scales (21, 24, 25). RT-induced acute dermatitis, though reversible in the vast majority of cases, can delay the overall radiotherapy treatment time, negatively influence the quality of life of patients, and require symptom management (16, 21, 24–27). Some factors amongst others that may affect the occurrence and severity of RD caused by RT are the irradiated body surface area and anatomy, the total dose and dose per fraction, the radiotherapy technology used, and concurrent treatment with systemic agents. Recent findings predict that vasodilation and angiogenesis contribute to radiation-induced dermatitis in post-menopausal RT breast cancer patients (24). Vasodilation and angiogenesis are related to the functional variations in oxygenation and perfusion to the region of interest.

Our hypothesis was that pre-RT vasodilation and angiogenesis contributed to RD in RT treated breast cancer patients; and tissue oxygenation measurements are directly related to these two physiological parameters that can be measured using a near-infrared imaging approach. From our current studies, we proved our hypothesis that the difference in tissue oxygenation contrast between the irradiated ipsilateral and the contralateral chest wall was a potential indicator of RD severity (grade 2 or lesser). This implies that there are inherent differences in breast’s tissue oxygenation in response to surgical removal of cancer (more so in mastectomy than lumpectomy) that potentially leads to a severe skin toxicity upon RT. Early identification of differences in these oxygenation differences pre-RT is essential to determine ways to predict extent of toxicity and guide interventional therapies, when used in conjunction with other dosimetric and non-dosimetric factors that assess RD risk.

Understanding the physiological changes after the completion of the RT effect was also performed in our study. While the severity of RD decreased during the one-month follow-up visit, the tissue oxygenation did not recover (or increase). In cases where we imaged during their third-month follow-up, the tissue oxygenation seems to have increased more distinctly from the last RT treated visit. This implies that the physiological recovery is slower than the visual RD grading as observed. Several patients observed an increase in RD severity after completion of their RT but were not clinically assessment until the one-month follow-up. This severity in RD could have lowered the tissue oxygenation even further and shown as a slower recovery during their follow-up visit. In future studies, the weekly imaging after completion of RT will need to be carried out to continuous monitor these oxygenation changes along with clinical assessment towards better correlations and understanding the underlying physiology.

## Conclusions

The objective of our studies was to derive correlations between tissue oxygenation and skin toxicity in breast cancer patients by monitoring their tissue oxygenation changes throughout their radiation therapy using a NIR-based imaging technique. The studies demonstrated significant changes in oxygen saturation (StO_2_) of the ipsilateral and contralateral chest wall and axilla during RT. These changes were evaluated to be negatively correlated to the development of radiation dermatitis (RD). It was also shown that pre-RT assessment of StO_2_ can potentially be part of the contributing factors toward risk assessment to determine if a patient is pre-disposed to adverse reactions during RT. Post-RT assessment of StO_2_ showed potential in providing additional sub-clinical information to monitor RD and tissue health after the completion of RT to assess complete extent of recovery. Future work will involve a larger focused subgroup of these RT treated breast cancer patients to develop threshold factors from pre-RT analysis that can predict the severity of RD along with non-clinical parameters and comorbidities. The effect of various clinical factors such as time between surgery and RT, chemotherapy used during surgery or RT, hormonal agents used during RT, and ointments used to tread RD severity. Our imaging approach will also be expanded to head and neck cancer patients to develop physiological and non-clinical parameter-based risk assessment in RD prediction, RD monitoring during RT, and physiological recovery rate post-RT

## Data Availability Statement

The raw data supporting the conclusions of this article will be made available by the authors, without undue reservation.

## Ethics Statement

The studies involving human participants were reviewed and approved by WIRB-A WIRB Copernicus Group Company. The patients/participants provided their written informed consent to participate in this study.

## Author Contributions

ER: The lead author to have performed all the imaging studies, developed study design, data analysis, and writing the manuscript. JM: Involved in data management and data analysis. RM: Was involved in complete statistical analysis along with the first author. KL: Involved in clinical visits and imaging studies along with the first author. CB: Involved as the clinical coordinator in the patient recruitment, and assistance during imaging studies. MR, MF and JP: Radiation oncologists who provided clinical assessments and feedback on each patient. JP also assisted in proposing image analysis and discussion of our results and manuscript submission. MC: Radiation oncologist who was the key investigator at Miami Cancer Institute responsible in the initial study design, IRB approvals, establishing the study, and assistance in the manuscript submission. WW: Biostatistician who assisted in all the statistical analysis and methods to implement for this study. AG: Corresponding author who led the entire study and the effort from proposing the idea, executing it, and completing this manuscript. All authors contributed to the article and approved the submitted version.

## Funding

This work was supported by the Coulter Seed Grant from FIU CEC-BME.

## Conflict of Interest

The authors declare that the research was conducted in the absence of any commercial or financial relationships that could be construed as a potential conflict of interest.

## Publisher’s Note

All claims expressed in this article are solely those of the authors and do not necessarily represent those of their affiliated organizations, or those of the publisher, the editors and the reviewers. Any product that may be evaluated in this article, or claim that may be made by its manufacturer, is not guaranteed or endorsed by the publisher.

## References

[1] Siegel RL Miller KD Fuchs HE Jemal A. Cancer Statistics, 2022. Cancer J Clin (2022) 72(1):7–33. doi: 10.3322/caac.21708 35020204

[2] Glennie DL. Integrating Sphere-Based Spectrally Constrained Total Diffuse Reflectance for In Vivo Quantification of Hemoglobin in Skin (2015). Available at: https://www.semanticscholar.org/paper/Integrating-sphere-based-spectrally-constrained-for-Glennie/b5cb9fd3f177412d7505becada28b773c3e72a93 (Accessed February 4, 2022).

[3] Yohan D Kim A Korpela E Liu S Niu C Wilson BC. Quantitative Monitoring of Radiation Induced Skin Toxicities in Nude Mice Using Optical Biomarkers Measured From Diffuse Optical Reflectance Spectroscopy. BioMed Opt Exp (2014) 5(5):1309–20. doi: 10.1364/BOE.5.001309 PMC4026905 24876997

[4] Chin LCL Cook EK Yohan D Kim A Niu C Wilson BC. Early Biomarker for Radiation-Induced Wounds: Day One Post-Irradiation Assessment Using Hemoglobin Concentration Measured From Diffuse Optical Reflectance Spectroscopy. BioMed Opt Exp (2017) 8(3):1682–8. doi: 10.1364/BOE.8.001682 PMC5480571 28663856

[5] Sunar U Quon H Durduran T Zhang J Du J Zhou C. Noninvasive Diffuse Optical Measurement of Blood Flow and Blood Oxygenation for Monitoring Radiation Therapy in Patients With Head and Neck Tumors: A Pilot Study. J BioMed Opt (2006) 11(6):064021. doi: 10.1117/1.2397548 17212544

[6] Nystrom J Geladi P Lindholm-Sethson B Rattfelt J Svensk AC Franzen L. Objective Measurements of Radiotherapy-Induced Erythema. Skin Res Technol (2004) 10(4):242–50. doi: 10.1111/j.1600-0846.2004.00078.x 15536655

[7] Nystrom J Svensk AC Lindholm-Sethson B Geladi P Larson J Franzen L. Comparison of Three Instrumental Methods for the Objective Evaluation of Radiotherapy Induced Erythema in Breast Cancer Patients and a Study of the Effect of Skin Lotions. Acta Oncol (2007) 46(7):893–9. doi: 10.1080/02841860701243087 17917821

[8] Abdlaty R. Hyperspectral Imaging and Data Analysis of Skin Erythema Post Radiation Therapy Treatment. Hamilton (ON: McMaster University (2016).

[9] Chin MS Siegel-Reamer L FitzGerald GA Wyman A Connor NM Lo Y. Association Between Cumulative Radiation Dose, Adverse Skin Reactions, and Changes in Surface Hemoglobin Among Women Undergoing Breast Conserving Therapy. Clin Transl Radiat Oncol (2017) 4:15–23. doi: 10.1016/j.ctro.2017.03.003 29594203 PMC5833900

[10] Chin MS Freniere BB Lo YC Saleeby JH Baker SP Strom HM. Hyperspectral Imaging for Early Detection of Oxygenation and Perfusion Changes in Irradiated Skin. J BioMed Opt (2012) 17(2):26010. doi: 10.1117/1.JBO.17.2.026010 22463042

[11] Chin MS Freniere BB Lancerotto L Lujan-Hernandez J Saleeby JH Lo YC. Hyperspectral Imaging as an Early Biomarker for Radiation Exposure and Microcirculatory Damage. Front Oncol (2015) 5:232. doi: 10.3389/fonc.2015.00232 26579490 PMC4620692

[12] Leiva K Robledo EA Beiner C Meyer B Murillo J Rodrigues MA. Asymmetry in Oxygenation Flow Patterns Between Irradiated and Contralateral Breast Tissues in Relation to Radiation Dermatitis. Proc SPIE 11618: Photon Dermatol Plast Surg (2021), 116180N. doi: 10.1117/12.2578781

[13] Ponticorvo A Rowland R Adelusi S Leproux A Wei R Kuo J. Using Spatial Frequency Domain Imaging (SFDI) to Quantify Physiological Changes of Patients Undergoing Radiotherapy for Breast Cancer Treatment. Proc SPIE 11211: Photon Dermatol Plast Surg (2020), 11211. doi: 10.1117/12.2547033

[14] Gibbons JD. Nonparametric Statistical Inference. New York: M. Dekker (1985).

[15] Hollander M Wolfe DA. Nonparametric Statistical Methods. New York: Wiley (1973).

[16] Wei J Meng L Hou X Qu C Wang B Xin Y. Radiation-Induced Skin Reactions: Mechanism and Treatment. Cancer Manag Res (2019) 11:167–77. doi: 10.2147/CMAR.S188655 PMC6306060 30613164

[17] Eltzschig HK Carmeliet P. Hypoxia and Inflammation. N Engl J Med (2011) 364(7):656–65. doi: 10.1056/NEJMra0910283 PMC3930928 21323543

[18] McGhee PB. Uf’s Proton Therapy Institute in Jacksonville Helps Doctors Train Their Sights on Cancer. Targeting Tumor (2006) 11(3):28–33.

[19] Mitin T Zietman AL. Promise and Pitfalls of Heavy-Particle Therapy. Am J Clin Oncol (2014) 32(26):2855–63. doi: 10.1200/JCO.2014.55.1945 PMC4152713 25113772

[20] Raine-Fenning NJ Brincat MP Muscat-Baron Y. Skin Aging and Menopause. Am J Clin Dermatol (2003) 4(6):371–8. doi: 10.2165/00128071-200304060-00001 12762829

[21] Behroozian T Milton L Li N Zhang L Lou J Karam I. Predictive Factors Associated With Radiation Dermatitis in Breast Cancer. Cancer Treat Res Commun (2021) 28:100403. doi: 10.1016/j.ctarc.2021.100403 34082363

[22] Liang X Bradley JA Zheng D. Prognostic Factors of Radiation Dermatitis Following Passive-Scattering Proton Therapy for Breast Cancer. Radiat Oncol (2018) 13(72). doi: 10.1186/s13014-018-1004-3 PMC5909216 29673384

[23] Kumar S Juresic E Barton M Shafiq J. Management of Skin Toxicity During Radiation Therapy: A Review of the Evidence. J Med Imaging Radiat Oncol (2010) 54(3):264–79. doi: 10.1111/j.1754-9485.2010.02170.x 20598015

[24] Alexopoulou E Katsila T Tolia M Tsoukalas N Leontsindis M Kyrgias G. An Exploratory Study of Radiation Dermatitis in Breast Cancer Patients. Anticancer Res (2018) 38:1615–22. doi: 10.21873/anticanres.12392 29491093

[25] Kole AJ Kole L Moran MS. Acute Radiation Dermatitis in Breast Cancer Patients: Challenges and Solutions. Breast Cancer (Dove Med Press). (2017) 2017(9):313–23. doi: 10.2147/BCTT.S109763 PMC5426474 28503074

[26] Primavera G Carrera M Berardesca E Pinnaró P Messina M Arcangeli GA. Double-Blind, Vehicle-Controlled Clinical Study to Evaluate the Efficacy of MAS065D (XClair™), a Hyaluronic Acid-Based Formulation, in the Management of Radiation-Induced Dermatitis. Cutan. Ocul. Toxicol (2006) 25(3):165–71. doi: 10.1080/15569520600860009 16980242

[27] Lam E Caitlin Y Wong G Popovic M Drost L Pon K. A Systematic Review and Meta-Analysis of Clinician-Reported Versus Patient-Reported Outcomes of Radiation Dermatitis. Breast (2020) 50:125–34. doi: 10.1016/j.breast.2019.09.009 PMC7375608 31563429

